# Impact of scaphoid non-union characteristics on healing outcomes

**DOI:** 10.1016/j.jpra.2025.10.028

**Published:** 2025-10-24

**Authors:** Raffael Labèr, Géraldine Lautenbach, Andreas Schweizer

**Affiliations:** Department for Hand Surgery, University Hospital Balgrist, Zurich, Switzerland

**Keywords:** Bone morphology, Hand surgery, Osteosynthesis, Pseudarthrosis, Scaphoid non-union

## Abstract

**Introduction:**

Scaphoid non-union remains a challenging clinical condition with the potential to result in long-term functional impairment. Identifying predictive factors for postoperative healing could improve treatment strategies and patient counselling. This study aimed to evaluate the potential impact of specific scaphoid non-union characteristics on healing time and the risk of persistent non-union following surgical intervention.

**Material and methods:**

We retrospectively analyzed 100 cases of surgically treated scaphoid non-unions between 2002 and 2020. The presence of scaphoid height difference, defect zone, and lateral intrascaphoid angle (LISA) and coronal intrascaphoidal angle (CISA) was documented. To determine associations with time to union and persistent non-union statistical testing used Mann-Whitney U, Spearman rank correlation, or Fisher’s exact test as appropriate. Multivariate linear regression was performed, and p-values were Bonferroni-corrected.

**Results:**

Of the 100 cases, 90 healed uneventfully, while 10 demonstrated persistent non-union at final follow-up. The median healing time was 73 days (interquartile range: 57–111 days). No statistically significant association was found between the assessed characteristics—scaphoid height difference, defect zone, LISA or CISA—and either time to union or persistent non-union.

**Conclusion:**

Our findings suggest that the four principal radiographic characteristics of scaphoid non-union do not significantly influence postoperative healing. These features may therefore be considered negligible in the prognostic assessment of surgical outcomes, supporting a more streamlined approach to preoperative evaluation.

## Introduction

Scaphoid fractures are typically caused by a fall onto an outstretched hand. They can be missed on X-rays and may sometimes progress to non-union.^1^ Even after adequate treatment, non-union occurs in 2 %–4 % of cases.^2^^,^^3^ Variables associated with non-union in previous studies include fracture site, fracture displacement, poor vascularity, carpal instability, heavy manual labor, and smoking.^4^^,^^5^

Analyzing different radiological parameters in the preoperative assessment to establish a prognosis for potential healing in scaphoid non-union surgery has been previously reported. Trabecular structure, sclerosis, or fragmentation observed in preoperative computed tomography (CT) scans has been analyzed, showing a correlation with histological healing capacity.^6^ CT scans provide a variety of information; therefore, as much of the available data as possible should be utilized.

The purpose of this study was to assess the influence of bony characteristics—specifically, the scaphoid height difference relative to the contralateral scaphoid, the defect zone, the lateral intrascaphoidal angle (LISA) and the coronal intrascaphoidal angle (CISA) measured in the injured wrist—on union time and the risk of persistent non-union following non-union surgery. We hypothesized that the aforementioned bony characteristics do not influence time to healing nor the risk of persistent non-union, as in our experience such bony features did not show an obvious association with outcome.

## Materials and methods

Patients were identified using CHOP (Swiss Classification of Operations) codes from electronic medical records. Data from patients who underwent surgery for scaphoid non-unions between 2002 and 2020 were reviewed retrospectively. Inclusion criteria were age ≥18 and preoperative CT scan. Exclusion criteria included previous failed scaphoid non-union surgeries or loss to follow up beyond six months in patients with only partial or no union. Scaphoid non-union was defined as the absence of union signs for six months post-injury or typical presentation of non-union in CT scans if the initial accident was not memorable. Patient informed consent was obtained, and the study was approved by the local ethics committee.

Surgical approach depended on the location and deformity of the scaphoid non-union, with procedures performed either from dorsal or palmar and primarily fixed using a headless compression screw. Bone grafts were utilized depending on defect size after debridement and the vascularity of the proximal pole. A vascularized distal radius graft was selected in the presence of compromised vascularity, while radial cancellous bone was preferred for adequately perfused scaphoids. When greater structural stability was needed, a graft from the iliac crest was utilized. Postoperative rehabilitation and follow up protocols were standardized. The wrist was immobilized with a split scaphoid cast for two weeks, followed by a circular scaphoid cast for another six weeks once swelling had sufficiently reduced. From eight to twelve weeks, a splint was recommended, and hand therapy was initiated with non-weight-bearing mobilization. From twelve weeks onwards, patients engaged in strengthening exercises. Follow-up assessments were conducted at two weeks (for suture removal) and at two, three, and six months, as well as at one year.

### Preoperative radiological assessment

**Figure 1:**
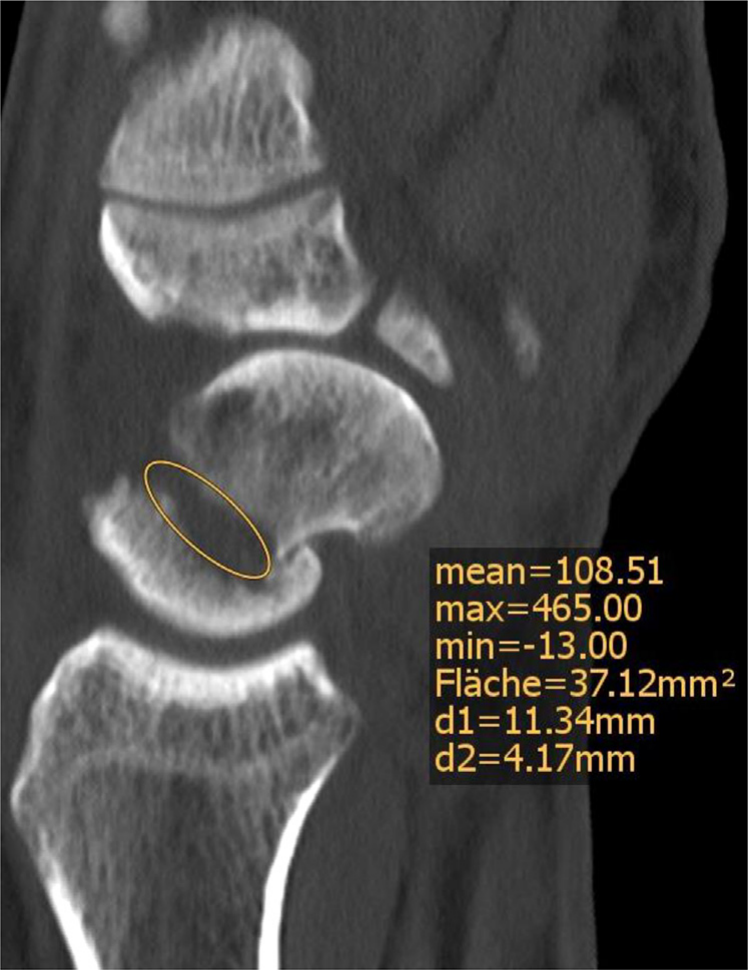
Measurement of the Defect zone sagittal view in a computed tomography and measurement of defect zone with an ellipse (mm^2^ = square millimetres).

The scaphoid height difference relative to the contralateral scaphoid was measured, while the defect zone, lateral intrascaphoidal angle (LISA) and coronal intrascaphoidal angle (CISA) were analyzed in the injured wrist. Height was measured from the proximal to distal pole in the coronal plane/posteroanterior (PA) view as previously described.^7^ The defect zone was measured using the Sanders view (*n* = 55) or in the sagittal plane/lateral view (*n* = 69) with an ellipse (Figure 1). Humpback deformity was assessed by measuring the LISA in the sagittal plane/lateral view and the CISA in the coronal view, drawing a perpendicular to the proximal and distal articular surfaces.^8^

### Postoperative radiological assessment

For union time analysis, two authors (R.L, G.L.) assessed healing. Union was defined as evidence of obliteration of more than half of the fracture line, while persistent scaphoid non-union was defined as <50 % union from six months onwards. However, the six-month threshold was not absolute, as healing in some cases extended to nine or twelve months, and these cases were not categorized as scaphoid non-union. In most cases (*n* = 95), a CT scan (computed tomography) was performed at the two month follow up, while in a few cases, an X-ray (*n* = 4) or both (*n* = 1) were used. Depending on previous follow up results, subsequent consultations included an X-ray, CT scan, or no imaging.

### Statistical analysis

To account for potential non-normal distribution of the data, we employed non-parametric statistical tests. Patient demographic characteristics are reported with mean and standard deviation or absolute and relative frequency as applicable. Other continuous variables are summarized by their median and interquartile range (IQR). Continuous variables were compared between groups using Mann-Whitney U tests and the distribution of categorical factors were compared between groups using Fisher’s exact tests. Associations between continuous parameters and the time until consolidation was assessed with spearman rank correlation tests. Associations of categorical risk factors with the time until consolidation were assessed with Spearman rank correlation tests or Mann-Whitney U tests, as applicable. Further risk analysis between categorical variables were conducted with Fisher’s exact tests. These p-values were corrected for the inflation of type I error rates (Bonferroni). A multivariate linear regression analysis was performed to identify parameters associated with the time to consolidation. A stepwise inclusion scheme was applied.

The analysis was conducted with SPSS (Version 28.0. Armonk, NY: IBM Corp). *P*-values below 0.05 were considered statistically significant.

## Results

A total of 100 patients with 100 cases were included, comprising 90 male and 10 female patients. The mean age at surgery was 29 years (standard deviation: 13). Of the patients, 23 were smokers, 53 were non-smokers, and the smoking status of 24 patients was unknown. Non-union occurred in the distal third in 6 % of cases, the middle third in 66 %, and the proximal third in 28 %. The calculated days (d) from accident to surgery resulted in a median of 240d (IQR: 109–459).

Scaphoid height, defect zone, and humpback deformity were assessed in all 100 cases. In 60 cases, imaging of the contralateral scaphoid was available, allowing height differences between the injured and contralateral sides to be calculated. The median scaphoid height difference to the contralateral scaphoid was 1 mm (millimetres, interquartile range [IQR]: 0–2), the median defect zone was 15 mm² (IQR: 9–25), the median LISA was 35° (IQR: 29–40), and the median CISA was 37° (IQR: 32–40).

Of the 100 cases, 90 healed uneventfully, whereas 10 had persistent non-union at final follow up, with a median healing time of 73 days (IQR: 57–111). Statistical analysis showed no association between scaphoid height difference, defect zone, LISA or CISA and either time to union (Table 1) or persistent non-union (Table 2).

**Table 1:** Risk factor analysis for time to union (Spearman’s rho).

Risk factors	Correlation Coefficient	*P*-value	(Bonferroni corrected)
Scaphoid height difference	0.045	0.747	1.0
Defect zone	−0.100	0.349	1.0
LISA	−0.037	0.729	1.0
CISA	0.019	0.862	1.0

LISA, lateral intrascaphoidal angle; CISA, coronal intrascaphoidal angle, significant (*P* < 0.05).

**Table 2:** Risk factor analysis for persistent non-union.

Risk factors	Healed	Not healed	*P*-value	*P*-value
	(mean (SD) or median (IQR))	(median (IQR))		(Bonferroni corrected)
Age (years)	28 (13)	32 (10)	0.182	1.0
Days accident until surgery	226 (107–424)	462 (211–3397)	0.065	0.390
Scaphoid height difference (mm)	1 (0–2)	0 (0–2)	0.777	1.0
Defect zone (mm^2^)	16 (9–25)	13 (9–15)	0.465	1.0
LISA (°)	35 (28–39)	37 (33–40)	0.401	1.0
CISA (°)	37 (31–40)	39 (33–40)	0.504	1.0

SD, standard deviation; IQR, interquartile range; mm, millimetres; mm^2^, square millimetres; LISA, lateral intrascaphoidal angle; °, degree, CISA, coronal intrascaphoidal angle; significant (*P* < 0.05).

## Discussion

The aim of this study was to analyze bone-specific characteristics, as determined by computed tomography (CT), that may be associated with prolonged healing time and persistent non-union. CT scans are the standard imaging modality for diagnosing acute carpal fractures.^9^ However, in cases of scaphoid non-union, CT imaging provides a wide range of valuable information about the pathology. It reveals bone morphology in two dimensions—and, if desired, even in three—offering the surgeon an enhanced visual understanding. Additionally, non-union-related features such as fragment dislocation and angulation can be clearly identified. Moreover, information about the quality of the remaining bone—such as absence of trabeculae, presence of sclerosis, or fracture of the proximal pole—can be obtained, offering insight into the potential healing capacity.^6^ These findings are used for treatment planning, including the surgical approach, type of graft, and method of fixation. They can also provide a prognosis regarding treatment success, which is highly valuable to both the patient and the surgeon. Therefore, analyzing the readily accessible information from CT scans to extract as much diagnostic value as possible is an appealing strategy. Several factors have already been examined, such as fragment dislocation, humpback deformity measured by LISA, height-to-length ratio, and dorsal cortical angle, in a smaller cohort of 63 patients.^10^ Thus, the aim of this study was to analyze the defect zone, the difference in scaphoid height compared to the contralateral side, the LISA and the CISA in a large patient cohort. Based on the authors’ clinical experience, no association had been observed between these variables and healing outcomes. Therefore, the hypothesis was formulated that the described parameters do not influence healing time or the risk of persistent non-union—an assumption that could be confirmed. These findings reinforce the conclusion that the examined variables do not affect healing duration or the risk of non-union and may therefore be considered clinically negligible. These results are consistent with the previous findings of Gvozdenovic^10^ and colleagues, who also investigated the LISA in a study involving only 63 cases. Moreover, only half of the cases they examined were true scaphoid non-unions (> 6 months), while the remaining were delayed unions (<6 months). Our findings stay in contrast with the findings of Grewal and colleagues,^11^ who reported that a greater humpback deformity was associated with slower union in acute scaphoid fractures; however, bony cysts showed no influence on healing time either.^11^

Explaining the observed findings is challenging; however, one possible explanation is that 49 % of cases (*n* = 49) underwent preoperative three-dimensional planning, leading to better scaphoid reduction. A retrospective study demonstrated improved accuracy in three-dimensional planning for scaphoid non-union and fracture surgeries, with mean three-dimensional angles improving from 25° (standard deviation: 6) to 7° (standard deviation: 3) compared to an improvement from 29° (standard deviation: 11) to 26° (standard deviation: 10) in cases without three-dimensional planning.^12^ Other contributing factors may include optimal debridement of the sclerotic zone and appropriate bone graft selection, though the impact of graft choice has been shown to be of minor significance.^13^ The limitations of this study include its retrospective design and the fact that, for the comparative analysis of scaphoid height differences in the 100 cases of scaphoid non-union, a contralateral image was available in only 60 cases. The sample size was determined by the number of cases that satisfied the inclusion criteria. Consequently, an effect equal to or greater than 1 can be detected by 80 % of the time; however, smaller effects may be overlooked. It is imperative that the results presented herein are interpreted with the requisite degree of caution and in accordance with the established conventions of scientific rigor. It is important to note that the present study examined a cohort 1.5 times the size of those previously reported. Further research is warranted to identify potential risk factors contributing to persistent non-union following surgical treatment.

## Level of evidence

IV.

## Funding

None.

## Ethical approval

Ethical approval for this study was obtained from local ethical commission (Ethikkommission Kanton Zürich, No. 2021-02485).

## Declaration of competing interest

None.
